# Quantitative Analysis of Protein Phosphorylations and Interactions by Multi-Colour IP-FCM as an Input for Kinetic Modelling of Signalling Networks

**DOI:** 10.1371/journal.pone.0022928

**Published:** 2011-07-29

**Authors:** Sumit Deswal, Anna K. Schulze, Thomas Höfer, Wolfgang W. A. Schamel

**Affiliations:** 1 Max Planck Institute of Immunobiology and Epigenetics, and Faculty of Biology, Biology III, University of Freiburg, Freiburg, Germany; 2 Spemann Graduate School of Biology and Medicine, Freiburg, Germany; 3 Research Group Modeling of Biological Systems, German Cancer Research Center and BioQuant Center, Heidelberg, Germany; 4 BIOSS Centre for Biological Signalling Studies, University of Freiburg, Freiburg, Germany; 5 Centre of Chronic Immunodeficiency (CCI), University Medical Center Freiburg, and University of Freiburg, Freiburg, Germany; Memorial Sloan Kettering Cancer Center, United States of America

## Abstract

**Background:**

To understand complex biological signalling mechanisms, mathematical modelling of signal transduction pathways has been applied successfully in last few years. However, precise quantitative measurements of signal transduction events such as activation-dependent phosphorylation of proteins, remains one bottleneck to this success.

**Methodology/Principal Findings:**

We use multi-colour immunoprecipitation measured by flow cytometry (IP-FCM) for studying signal transduction events to unrivalled precision. In this method, antibody-coupled latex beads capture the protein of interest from cellular lysates and are then stained with differently fluorescent-labelled antibodies to quantify the amount of the immunoprecipitated protein, of an interaction partner and of phosphorylation sites. The fluorescence signals are measured by FCM. Combining this procedure with beads containing defined amounts of a fluorophore allows retrieving absolute numbers of stained proteins, and not only relative values. Using IP-FCM we derived multidimensional data on the membrane-proximal T-cell antigen receptor (TCR-CD3) signalling network, including the recruitment of the kinase ZAP70 to the TCR-CD3 and subsequent ZAP70 activation by phosphorylation in the murine T-cell hybridoma and primary murine T cells. Counter-intuitively, these data showed that cell stimulation by pervanadate led to a transient decrease of the phospho-ZAP70/ZAP70 ratio at the TCR. A mechanistic mathematical model of the underlying processes demonstrated that an initial massive recruitment of non-phosphorylated ZAP70 was responsible for this behaviour. Further, the model predicted a temporal order of multisite phosphorylation of ZAP70 (with Y319 phosphorylation preceding phosphorylation at Y493) that we subsequently verified experimentally.

**Conclusions/Significance:**

The quantitative data sets generated by IP-FCM are one order of magnitude more precise than Western blot data. This accuracy allowed us to gain unequalled insight into the dynamics of the TCR-CD3-ZAP70 signalling network.

## Introduction

Inducible protein-protein interactions and post-translational modifications are the major mode of cellular communication and are responsible for cellular decisions such as cell proliferation, differentiation, survival or death [1], [2]. Despite their importance, precise quantitative measurements of these interactions and modifications remain difficult. The most commonly used protein analysis method is immuno-precipitation (IP) followed by SDS-PAGE and Western blotting (IP-WB). This technique has provided invaluable insight into signalling pathways; however, it is neither very quantitative nor high-throughput. This limits the mechanistic analysis of signalling modules using mathematical tools [3] for two reasons. First, the rather large experimental error of standard IP-WB makes it difficult to distinguish between competing alternative models. Second, the parameterization of mathematical models often requires quantitative information, such as the fraction of phosphorylated molecules in the total pool of a protein.

In contrast, flow cytometry (FCM) accurately measures fluorescence intensities over several orders of magnitude and therefore is perfectly suited to generate quantitative data. Further, it can currently simultaneously measure up to 17 different fluorescence channels in a high-throughput manner [4]. In cytometric bead arrays [5], [6] or IP measured by FCM (IP-FCM) [7], [8], antibody-coupled beads are utilized to capture the protein of interest from cellular lysates. After IP, the beads are stained with a fluorophore-coupled antibody to quantify the amount of this protein by one-colour FCM. Thus, this methodology allows, for example, determining the level of cytokines in cellular supernatants [9], [10]. However, to retrieve precise data on phosphorylations [6], [11] or interaction partners [7] it is necessary to also measure the amount of the protein directly captured on the beads, for normalization reasons. This is not possible with one-colour IP-FCM.

WB can generate relative data, such as that upon stimulation the interaction between two proteins is increased by a factor of 20. Absolute values, such as 4 molecules of protein X are bound per protein Y, are difficult - and in many cases impossible - to determine by WB. One would need a defined reference amount of a certain protein or phosphorylation site (in µg), to apply to the same SDS-PAGE as the sample to be measured [12], [13], [14]. Such reference proteins or phospho-proteins might be difficult to obtain and the data suffer from the rather poor quantitation with WB.

Here, we exploit the feature of flow cytometers to simultaneously measure several fluorescence channels in a high-throughput manner. By doing so we have extended one-colour IP-FCM to a multi-colour technology platform to generate extremely precise protein data.

In this study we used membrane-proximal modules of the T-cell antigen receptor (TCR-CD3) intracellular signalling network, a system for which many mechanistic details are known and reagents are available. The TCR-CD3 is expressed on T-cells and consists of TCRαβ, CD3εγ, CD3δε and CD3ζζ dimers [15], [16], [17]. Upon stimulation, this complex is phosphorylated by kinases of the Src family, such as Lck, on tyrosine residues of the CD3 subunits [18], [19], which then serve as docking sites for the kinase ZAP70 [15], [18], [20]. One TCR-CD3 contains 10 binding sites for ZAP70 and thus could bind simultaneously to 10 ZAP70 molecules [21]. Consequently, ZAP70 itself is phosphorylated at tyrosine 319 (Y319) in the interdomain B and at Y493 in the kinase domain [22]. The crystal structure of ZAP70 [23] suggests, that in its unphosphoryated form ZAP70 is in an autoinhibitory state due to the binding of the tandem SH2 domains to the kinase domain. Phosphorylation at Y319 by Lck may release the inhibitory conformation [24], so that Y493 can be phosphorylated. Indeed, Lck kinase activity [25] and the presence of Y319 [26] are required for Y493 phosphorylation. Thus, phosphorylation of Y319 is a prerequisite for Y493 phosphorylation. Most likely, Y493 is phosphorylated by autophosphorylation [27], although older reports have suggested that Y493 can be phosphorylated by Lck [25], [28].

Full activity of ZAP70 requires phosphorylation at Y493 [25], which is located in the activation loop of the kinase domain. Active ZAP70 phosphorylates the adaptor protein LAT, which in turn activates downstream signalling pathways, such as the Ras/Erk pathway [29]. Pervanadate irreversibly oxidizes the catalytical center of tyrosine phosphatases, and thus inactivates them [30]. Here we use treatment of cells with pervanadate [31], [32] as a surrogate for antigen-binding to the TCR-CD3 complex, as it leads to phosphorylation of the CD3 subunits, recruitment and phosphorylation of ZAP70.

## Results

### Development of multi-colour IP-FCM

**Figure 1:**
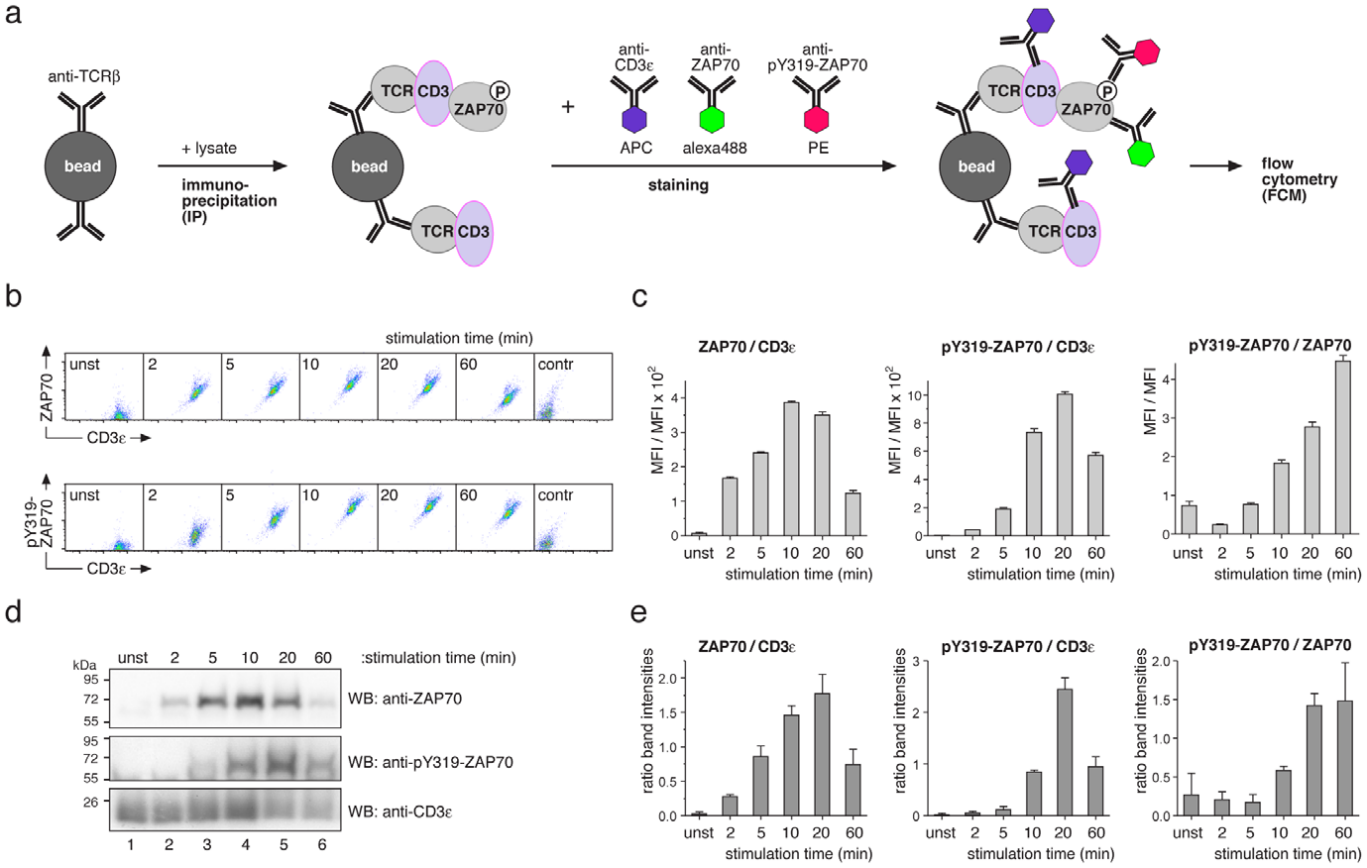
Three-colour IP-FCM. (**a**) Anti-TCRβ-coupled latex beads are used for immuno-precipitation of the TCR-CD3 complex from cell lysates. Purified proteins are stained with flourophore-conjugated antibodies recognizing CD3ε, ZAP70 and phosphorylated ZAP70 at position Y319. Fluorescence of the beads is measured by FCM. (**b**) Murine 2B4 T-cells were stimulated with 5 mM pervanadate for the indicated time points. After lysis three-colour IP-FCM was performed. Dot plots of CD3ε, ZAP70 and pY319-ZAP70 intensities are shown. As a staining control (contr), uncoupled beads were used. (**c**) The experiment from (b) was done in triplicates and the ratios of two geometric mean fluorescence intensities (MFI) after background subtraction are shown in the arbitrary units. (**d**) Cells were stimulated as in (b), the TCR-CD3 was immuno-precipitated and proteins subjected to SDS-PAGE and WB using the antibodies indicated. (**e**) The experiment from (d) was done in triplicates and band intensities were determined. The ratios of two intensities are shown. Mean ± s.e.m. values are graphed in (c) and (e).

To expand IP-FCM to a multi-colour technique, we stimulated the murine 2B4 T-cell line [33], [34] with the phosphatase inhibitor pervanadate at multiple time points. After cell lysis, the TCR-CD3 complex was purified with anti-TCRβ-coupled beads. We used polystyrene latex beads, which have a defined size and low autofluorescence. The kinetics of ZAP70 recruitment and phosphorylation at Y319 was profiled by staining of the protein-bound beads with anti-ZAP70-alexa488 (green) and anti-phospho-Y319-ZAP70-PE (anti-pY319-ZAP70-PE, red). In addition, the level of CD3ε was quantified as an internal control for the amount of purified TCR-CD3 using anti-CD3ε-APC (purple, **Fig. 1a**). Each stimulation was performed in triplicates and more than 60 data points were recorded using an autosampler sending the beads to a Gallios flow cytometer (Beckman Coulter) in a format suitable for high-throughput measurement. We simultaneously quantified the amount of ZAP70, pY319-ZAP70 and CD3ε (**Fig. 1b**). The mean fluorescence intensities (MFIs) of the anti-ZAP70 and anti-pY319-ZAP70 stains were normalized by the MFI of the anti-CD3ε stain (**Fig. 1bc**). In addition, the ratio of the MFIs of the anti-ZAP70 and anti-pY319-ZAP70 stains is also shown (**Fig. 1bc**).

We found that ZAP70 was recruited with faster kinetics (maximum at 10 min) than its phosphorylation (maximum at 20 min), resulting in an increase in the relative amount of pY319-ZAP70 up to 60 min (**Fig. 1c**). Surprisingly, the ratio of pY319-ZAP70 to ZAP70 first decreases (2 min stimulation) and then increases. The reason for this is unknown and will be addressed later (see below).

A concern that arises with the introduction of a new technology is the comparative performance with an existing technology. Hence, we compared the multi-colour IP-FCM experiment with standard IP-WB (**Fig. 1d, e**). Indeed, a similar kinetics of ZAP70 recruitment and phosphorylation was obtained. However, IP-FCM showed a smaller standard deviation. A statistical analysis demonstrated that the percent coefficient of variation (%cv) in IP-FCM ranged from 4.0 to 7.2, whereas in IP-WB it was 31 to 86 (**Table S1**). Thus, IP-FCM was approximately 10 times more precise than IP-WB, although the Luminescent Image Analyzer LAS-4000 (Fujifilm Life-Science) was used for WB quantification. Testing the sensitivity of IP-FCM, we could detect ZAP70 recruitment with 0.4 µg/ml total protein in the lysate (**Fig. S1**).

Further validation was done by staining of the beads with each antibody separately (see below figure 5) and by inhibiting the Src kinase before pervanadate treatment. Src kinases phosphorylate CD3 and inhibition should abolish ZAP70 recruitment. Indeed, recruitment of ZAP70 to the TCR and ZAP70 phosphorylation was reduced when cells were pre-treated with the Src kinase inhibitor PP2 (**Fig. S2**). We also show that the anti-ZAP70 antibody can recognize ZAP70 of resting and stimulated cells to a similar extent (**Fig. S3**).

**Figure 2:**
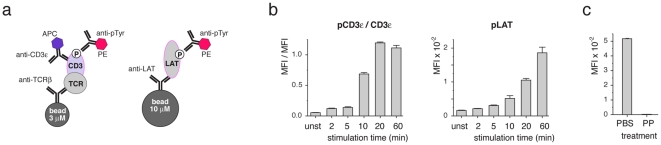
Expansion of IP-FCM. (**a**) Scheme of a two-plexed, two-colour IP-FCM quantifying the phosphorylation of CD3 and LAT. Anti-TCRβ- and anti-LAT-coupled beads are mixed for IP and distinguished in FCM according to their size. (**b**) 2B4 cells were stimulated as in figure 1 and the two-plexed, two-colour IP-FCM was performed. The ratio of the MFIs of phospho-CD3ε and CD3ε and the MFI of phospho-LAT are shown. (**c**) The experiment was done as in (b) with the inclusion of 0.1 M phenyl-phosphate (PP) during the anti-phospho-tyrosine staining step. The MFI of phosphorylated CD3 at 10 min is shown. Mean ± s.e.m. values are displayed.

In order to further extend the multi-colour IP-FCM approach, we used two different latex beads that can be distinguished after FCM by electronic gating according to their different size (**Fig. S4**). 3 µm beads were coupled to anti-TCRβ and 10 µm beads to anti-LAT antibodies. A 1∶1 mixture of the beads was added to lysates of pervanadate-stimulated 2B4 cells for IP. Beads were stained with anti-phospho-tyrosine-PE and anti-CD3ε-APC antibodies (**Fig. 2a**) and analysed by FCM (**Fig. 2b**). Gating on the 3 µm beads, the MFI of the PE fluorescence corresponded to the amount of phosphorylated TCR-CD3. This intensity was normalized to the MFI of the anti-CD3ε stain (**Fig. 2b**, left panel). Gating on the 10 µm beads allowed quantifying the amount of phospho-LAT (right panel). As expected, phosphorylation of LAT occurred with a delayed kinetics compared to the one of TCR-CD3. Again the average %cv was small (5.6, 3.4 and 11 for phospho-TCR-CD3, TCR-CD3 and phospho-LAT, respectively).

To show the specificity of the anti-phospho-tyrosine antibody, phenyl phosphate was included in the staining step, which efficiently competed with the phospho-tyrosines for binding to the PE-conjugated anti-phospho-tyrosine antibody, reducing the fluorescence intensity of the beads (**Fig. 2c**). Also in this experiment, the standard deviation was small (average %cv = 0.72). Tyrosine phosphorylated proteins could also be identified by anti-phosphotyrosine IP and staining for different proteins in the same sample, using antibodies with distinct fluorophores (**Fig. S5**).

**Figure 3:**
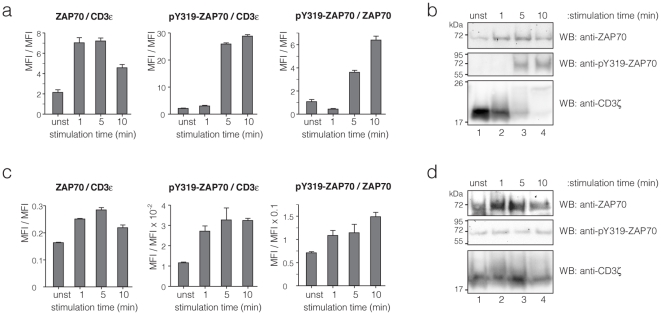
IP-FCM and IP-WB measurement using primary T-cells. (**a**, **b**) Splenocytes from OT-1 TCRαβ transgenic mice were stimulated with 5 mM pervanadate and lysed at different time points. The lysates were divided in two parts and used for three-colour IP-FCM as in figure 1 (**a**) or for IP-WB measurements (**b**). In (**a**) the ratios of two MFIs after background subtraction from IP-FCM are shown. Error bars represent mean +/− s.e.m. (**c**, **d**) Splenocytes from OT-1 mice were stimulated with 100 nM OVA peptide-MHC tetramer ligand and lysed at different time points. The lysates were divided in two parts and used for three-colour IP-FCM as in figure 1 (**c**) or for IP-WB measurements (**d**). In (**c**) the ratios of two MFIs after background subtraction from IP-FCM are shown. Error bars represent mean +/− s.e.m.

Next, we tested whether we could monitor the kinetics of ZAP70 recruitment and phosphorylation in primary, naïve T-cells using pervanadate as well as the cognate ligand, MHC-peptide (MHCp), as the stimulus. To this end, splenocytes from OT-1 TCRαβ transgenic mice [35] were stimulated with 5 mM pervanadate or 100 nM MHCp tetramers for different time points and the three-colour IP-FCM of figure 1 was performed (**Fig. 3a and c**). The comparison demonstrates that pervanadate resulted in a stronger stimulation than MHCp tetramers and that the kinetics of ZAP70 recruitment to the TCR-CD3 was similar in both stimulation conditions. However, TCR-CD3-bound ZAP70 is phosphorylated faster at Y319 upon tetramer stimulation (**Fig. 3c**), compared to pervanadate stimulation (**Fig. 3a**). The results were confirmed by IP-WB (**Fig. 3** **b** and **d**).

From unstimulated to maximally stimulated 2B4 cells the increase in ZAP70 association to the TCR-CD3 complex was 60-fold (**Fig. 1c**), whereas it was 3.4-fold in the primary cells. This could indicate, that some ZAP70 was associated with the TCR-CD3 independent of stimulation in primary cells as described [36].

**Figure 4:**
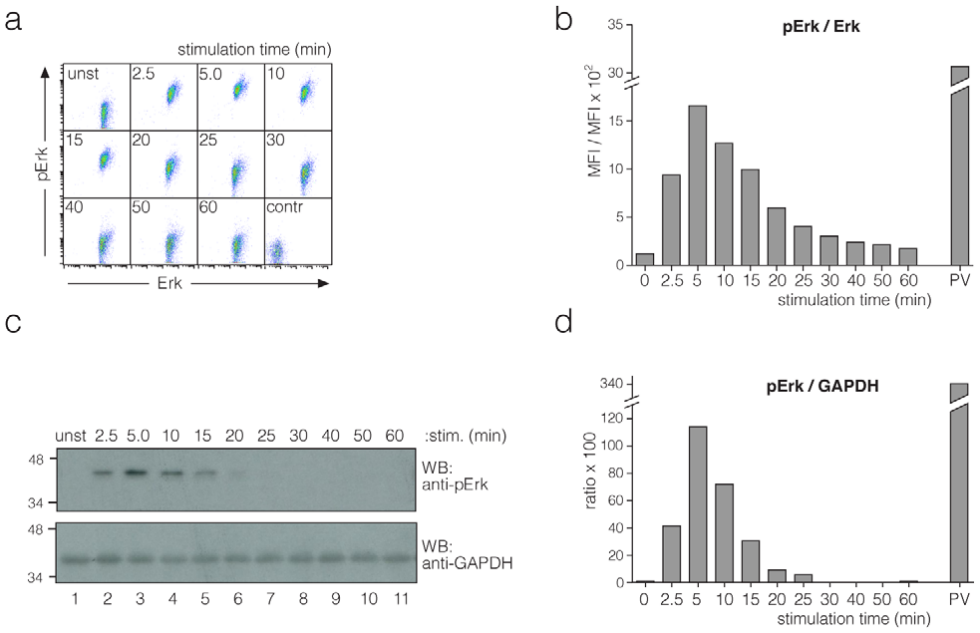
Quantification of phospho-Erk. 2B4 cells were stimulated with anti-CD3 antibodies for different time points or with pervanadate for 5 min. Two-colour IP-FCM purifying Erk and staining with anti-Erk-alexa488 and anti-phospho-Erk-alexa647 was done. Dot plots (**a**) and the ratio (**b**) of the MFIs are shown. The control in (a) is as in figure 1b. (**c**) Lysates from (a) were separated on a 12% SDS-PAGE. The WB membrane was developed using anti-phospho-Erk and anti-GAPDH antibodies as a loading control. Signal intensities were determined using the chemo-luminescence detection system. (**d**) The phospho-Erk signal intensity was normalized with respect to that of GAPDH.

Finally, the kinetics of Erk phosphorylation normalized to the total amount of Erk was quantified by two-colour IP-FCM (**Fig. 4a and b**). In this case, the antibodies only recognized unfolded Erk, but not the native protein. Thus, a slightly different experimental protocol was set up (**Fig. S6**). As in a WB experiment (**Fig. 4c and d**), maximum phosphorylation occurred at 5 min and then decayed. In this comparison IP-FCM was more sensitive than IP-WB. As expected, pervanadate stimulation resulted in higher phospho-Erk levels than anti-CD3 stimulation. Further comparison with the commercial one-colour bead-based BioPlex kit showed that, in contrast to the kit, our assay was able to generate normalized values, but was less sensitive (**Fig. S7**). The higher sensitivity of BioPlex kit might be because of use of biotin-labelled primary antibody and PE-labelled streptavidin for staining. In a multicolour IP-FCM use of biotin labelled primary antibodies and PE-labelled streptavidin is of limited use, as each antibody needs to be stained with a separate colour.

### Absolute quantification

The values of the y-axis in figure 1 are the ratios between two different fluorescence intensities, and as such have no biological counterpart. As in WB experiments, only a relative increase from sample 1 to sample 2 can be obtained. For example, we calculated that 60-fold more ZAP70 is bound per TCR-CD3, if the unstimulated sample is compared to the 10 min stimulated one (**Fig. 1c**), but we do not know how many ZAP70 molecules are bound per TCR-CD3 complex. However, IP-FCM offers a straightforward, easy-to-use protocol to determine absolute protein numbers, as follows.

**Figure 5:**
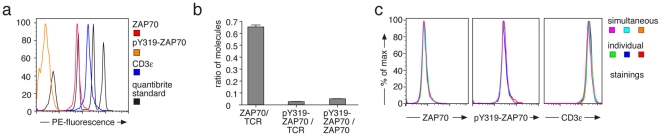
Generation of absolute values by IP-FCM. (**a**) 2B4 T-cells were pervanadate-stimulated for 10 min and lysed. IP was done with anti-TCRβ coupled latex beads and the beads were individually stained with saturating conditions of anti-ZAP70-PE (red), anti-pY319-ZAP70-PE (orange) and anti-CD3ε-PE (blue) antibodies. PE-labelled Quantibrite beads (black) were measured along with the samples to generate a standard curve of MFI versus PE molecules per bead. (**b**) From the standard curve, PE molecules per bead for the IP-FCM samples were determined in a triplicate experiment. The number of ZAP70 molecules and pY319-ZAP70 molecules per TCR-CD3 and the number of pY319-ZAP70 per ZAP70 are shown. Mean ± s.e.m. values are displayed. (**c**) 2B4 cells were pervanadate-stimulated for 20 min and lysed. IP was done with anti-TCRβ coupled latex beads and the beads were stained either with anti-ZAP70-alexa488, anti-pY319-ZAP70-alexa647 and anti-CD3ε-APC antibodies together (simultaneous, three colour IP-FCM) or with one antibody at a time (individual). Histograms to compare these stainings are shown for each of the antibodies. The experiment was done in triplicate as indicated by the histogram colours.

To measure absolute values, the TCR-CD3 was immunoprecipitated from lysates of 10 min pervanadate stimulated T-cells. The beads were split into three aliquots and individually stained with saturating conditions of anti-CD3ε-PE, anti-ZAP70-PE and anti-phospho-ZAP70-PE antibodies each with one PE fluorophore per antibody (**Fig. 5a**). Saturating conditions (that we tested experimentally, data not shown) were important for the quantification, to ensure that each molecule of CD3ε, ZAP70 or phospho-ZAP70 was bound by one antibody and thus labelled by exactly one PE fluorophore. Samples were measured by flow cytometry along with calibration beads with a defined number of PE molecules per bead (**Fig. 5a**, black lines), that were used to generate a standard curve of PE-MFI versus PE molecules per bead (**Fig. S8**). From the standard curve, we calculated that at 10 min of stimulation 16,000±500 CD3ε, 5,300±100 ZAP70 and 220±10 phosphorylated ZAP70 molecules were bound per bead. Finally, these data were used to calculate that 0.65±0.02 ZAP70 molecules were bound per TCR-CD3 complex (**Fig. 5b**), considering that each TCR-CD3 has two CD3ε subunits [16]. Of those ZAP70, 4.2±0.2% were phosphorylated at Y319, so that 0.028±0.02 pY319-ZAP70 molecules were bound per TCR-CD3 (**Fig. 5b**). The amount of ZAP70 per TCR-CD3 was lower than expected, since each TCR-CD3 can theoretically bind up to 10 ZAP70 molecules. Thus, these data are valuable information for our modelling approach (see below).

We excluded that the low ZAP70/TCR-CD3 ratio was a result of ZAP70 dissociation from the TCR-CD3 complex during the IP and staining steps of the IP-FCM procedure (**Fig. S9**). However, some ZAP70 could have been lost at cell lysis.

For the modelling we wanted to combine the kinetic data from figure 1 (three colour IP-FCM) with the absolute data from figure 5 (one colour IP-FCM). Thus, we had to show that simultaneous staining of the beads with three antibodies resulted in the same MFI as when the stainings were done individually. To this end, we stained the TCR-CD3-ZAP70-bound beads with the three fluorophore-conjugated antibodies in one tube, as in figure 1, or we split the beads in three equal aliquots after IP and performed the stainings individually. Similar staining intensities were obtained for each antibody in either procedure (**Fig. 5c**). This shows that there was no steric hindrance among the staining antibodies to bind to their respective epitopes and that the two data sets can be combined in one model.

### IP-FCM provides an accurate basis for a mechanistic understanding

**Figure 6:**
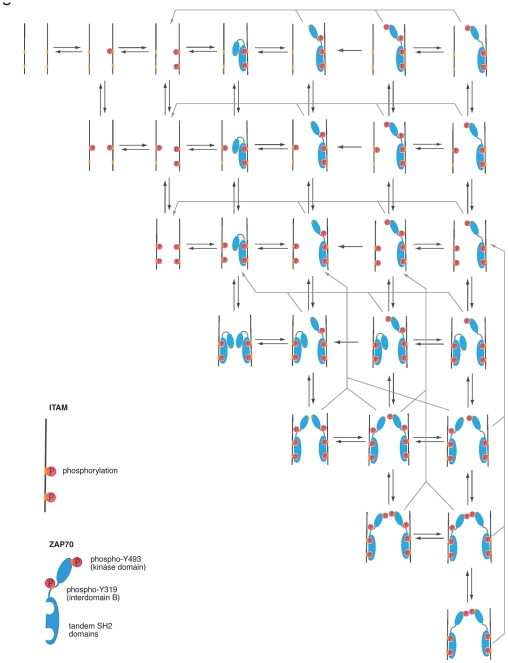
Mathematical model for early signalling events at the TCR. The model considers the phosphorylation of ITAMs by Lck, the binding of ZAP70 with its tandem SH2 domains to doubly phosphorylated ITAMs, the phosphorylation of Y319 of ZAP70 by Lck and of Y493 of ZAP70 by transphosphorylation. For the transphosphorylation two ZAP70 molecules both being in the opened conformation (phospho-Y319) have to be in close proximity. Therefore, the basic unit of the model is a pair of opposite ITAMs, as found in the paired CD3 and ζ chains. The scheme shows the possible configurations of the two ITAMs and bound ZAP70 molecules in the model, together with the reaction steps connecting the different states. Note that the two opposing ITAMs are treated as indistinguishable, so that it does not matter, for example, to which ITAM a ZAP70 molecule is bound. Likewise, the two phosphorylation sites within an ITAM are treated, for simplicity, as having identical properties. The differential equations governing the time evolution of the different states are given in Materials and methods.

The accurate IP-FCM measurements unequivocally demonstrated that the fraction of pY319-ZAP70 at the TCR-CD3 complex decreases in the first minutes of stimulation with pervanadate and exceeds the level seen in unstimulated cells only at times larger than 5 minutes (**Fig. 1c**). This phenomenon cannot be inferred from IP-WB, because WB data exhibit large measurement errors (**Fig. 1e**). Although clearly significant in the IP-FCM measurements, this initial decrease of the pY319-ZAP70/ZAP-70 ratio may appear surprising, because stimulation actually induces the phosphorylation of ZAP70. To provide a mechanistic rationale for this observation, we developed a mathematical model of the initial phosphorylation and protein recruitment steps at the TCR (**Fig. 6**). The model describes the core unit of TCR-CD3 signal transduction at the cytoplasmic tails of the TCR-CD3 complex, a pair of opposite ITAMs [20]. The model assumes that each of the ITAMs can be phosphorylated randomly on its two tyrosine residues by Lck. Then ZAP-70 binds to the phosphorylated ITAMs and is phosphorylated by Lck at Y319 [26], [37]. The resultant opening of the ZAP70 structure allows trans-autophosphorylation of two opposite ZAP70 at Y493, achieving full activity of the kinase [25]. Thus, a single ITAM can occur in seven different states (**Fig. 6**), resulting in 49 possible states of the ITAM pair. Each reaction step was modelled with first-order mass action kinetics, with the active Lck concentration and the concentration of the abundant free ZAP70 considered constant.

**Figure 7:**
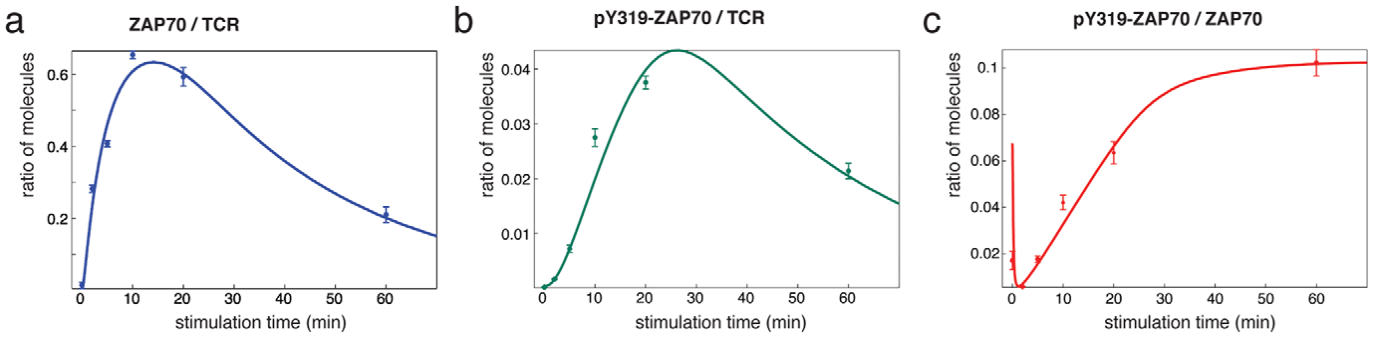
The mathematical model accounts for the experimentally observed kinetics of ZAP70 recruitment and phosphorylation. Least-square fitting of the differential equation system given in material and methods to the experimental data accurately reproduces the time courses of ZAP70 bound to the TCR-CD3 (**a**), pY319-ZAP70 bound to the TCR-CD3 and (**b**) the ratio of pY319-ZAP70 to ZAP70 both bound to CD3 (**c**). Data points are reproduced from figure 1c. The graphs are derived from the mathematical model.

Fitting this model to the IP-FCM data, we could match the data closely (**Fig. 7a, b and c**). In particular, the initial drop in the pY319-ZAP70/ZAP70 ratio at the TCR-CD3 complex was achieved (**Fig. 7c**) and the model suggests the following mechanistic interpretation. Under resting conditions, a small fraction of ITAMs is already phosphorylated and has ZAP70 bound. Part of this bound ZAP70 is phosphorylated at Y319. After stimulation with pervanadate, unphosphorylated ZAP70 is recruited from the cytoplasm to newly phosphorylated ITAMs. Thus, immediately after stimulation, there is more unphosphorylated than phosphorylated ZAP70 bound to the TCR, causing the drop in the bound pY319-ZAP70/ZAP70 ratio. Subsequently, this newly bound ZAP70 gets phosphorylated on Y319 by Lck, which lets the pY319-ZAP70/ZAP70 ratio rise again.

**Figure 8:**
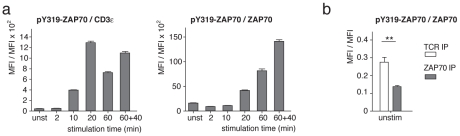
Model validation. (**a**) An experiment as in figure 1 was performed, including a 60 min stimulation, in which pervanadate was applied at time 0 and the stimulation was stopped at time 60. A second 60 min stimulation was done, in which pervanadate was applied at time 0 and again at time 20 and the stimulation was stopped at time 60 (60+40). (**b**) Unstimulated 2B4 T-cells were lysed and TCR-CD3 complexes or total ZAP70 were immunoprecipitated using anti-TCRβ- or anti-ZAP70-coupled latex beads. Beads were stained with anti-pY319-ZAP70-PE and anti-ZAP70-alexa488 antibodies and measured by FCM as in figure 1. The ratio of pY319-ZAP70 to ZAP70 is displayed**: p<0.05. Mean ± s.e.m. values are displayed.

The model could only be fitted to the data, if one assumed that phosphatase activity was regained after longer times of pervanadate treatment. Although pervanadate irreversibly inhibits phosphatases [30], it might be quenched with time and thus newly synthesized phosphatases might be active at longer stimulation times. To verify this prediction of the model, we repeated the experiment shown in figure 1 and added pervanadate at time zero and again after 20 min. Indeed, an increase in pY319-ZAP70 per TCR or per ZAP70 was detected when compared to one single dose of pervanadate at time zero (**Fig. 8a**). This shows that some phosphatase activity is present at longer stimulation times, validating the model.

Furthermore, the model predicted that in the unstimulated cells the amount of pY319-ZAP70 per ZAP70 is higher in the TCR-CD3-bound fraction than in the non-bound fraction. To test this prediction experimentally, we performed anti-TCRβ or anti-ZAP70 IPs from resting T-cells (**Fig. 8b**). Indeed, the ratio of pY319-ZAP70 per ZAP70 was significantly higher for the TCR-CD3-bound ZAP70 (anti-TCRβ IP) compared to the total fraction of ZAP70 (anti-ZAP70 IP).

### Phosphorylation of ZAP70 at Y319 precedes the one at Y493

**Figure 9:**
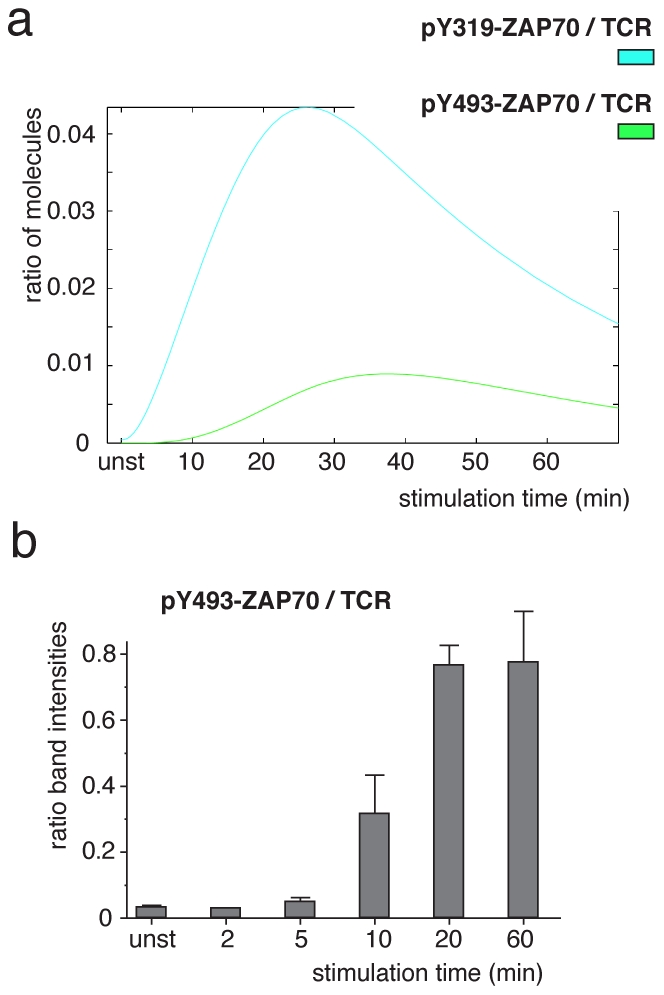
ZAP70 phosphorylation at Y493. (**a**) Using the model, the phosphorylation kinetics of ZAP70 at Y319 and Y493 was simulated and plotted. Phosphorylation at Y493 was delayed with respect to Y319. (**b**) 2B4 T-cells were stimulated with 5 mM pervanadate for the indicated time points. After lysis the TCR-CD3 was immuno-precipitated and proteins subjected to SDS-PAGE and WB using anti-pY493-ZAP70 and anti-CD3ζ antibodies. The experiment was done in triplicates and band intensities were determined. The ratio of pY493-ZAP70 to CD3ζ is shown in arbitrary units. Mean ± s.e.m. values are shown.

Using the mathematical model we could predict the phosphorylation kinetics of Y493, which we could not measure by the IP-FCM method due to technical reasons (data not shown). We therefore did not use information on Y493 phosphorylation for model fitting. When simulating the model, we observed that Y493 phosphorylation rose more slowly and persisted longer than Y319 phosphorylation (**Fig. 9a**). To test the model, we measured the phosphorylation kinetics of Y493 by IP-WB and, qualitatively, found the predicted delay in relation to the Y319 phosphorylation (**Fig. 9b**). This is in line with earlier suggestions that phosphorylation at Y319 is a prerequisite for phosphorylation at Y493 [24], [26].

In summary, the precise and absolute data generated by IP-FCM provided an accurate basis for parameterizing a mathematical model of early TCR phosphorylation and protein recruitment events (**Fig. S10**). This model allowed a detailed mechanistic reconstruction of the dynamics of these processes.

## Discussion

Here, we improved one-colour IP-FCM to develop a powerful technology platform for the high-throughput generation of multidimensional and quantitative data that serves as a new input for quantitative analysis. As a pertinent example, we showed its application to mathematical modelling of early signalling events in T-cell activation.

The main advantages of multi-colour IP-FCM are: (i) precise protein data with a high dynamic range, (ii) normalized data on protein phosphorylations and interactions, (iii) multiple parameters quantified simultaneously using two bead sizes and multiple colours, (iv) absolute values, in contrast to relative ones, can be obtained, (v) time effective, (vi) adaptable to a 96-well format for the generation of large data sets, (vii) except for a flow cytometer no special equipment is needed, and (viii) the assay can be run at native or denaturing conditions, depending on the availability of antibodies.

For IP-FCM, 5000 beads were measured and the geometric MFI was taken, in contrast to WB where only one quantification per point was done. This might contribute to the smaller error of IP-FCM, in addition of uneven WB transfer [38]. Furthermore, the high-throughput nature of IP-FCM easily allows measurement of more replicas than triplicates. This is very limited in IP-WB, and efforts are undertaken to develop SDS-PAGE and WB systems in which large numbers of samples can be applied [39].

We used PE-labelled calibration beads to obtain absolute values. Since defined FITC-labelled beads are also available, one could retrieve absolute values from two-colour IP-FCM.

High specificity of the stainings is reached by the “sandwich assay” nature of IP-FCM. Very few antibodies are truly monospecific, the majority also bind to at least one other cellular antigen. The sandwich assays achieve superb selectivity without the size fractionation afforded by WB, because the specificities of two different antibodies are exploited. Hence IP-FCM is fundamentally more specific compared to assays where only one antibody is employed, such as intracellular FCM staining. IP-FCM might also be used for quantification of other stimulus-induced events, as e.g. ubiquitinylation, methylation or proteolysis; and for identification of stimulus-specific changes in subcellular localization by cell fractionation prior to IP.

However, IP-FCM neither yields information on the protein size as does IP-WB, nor on individual cells as does intracellular staining for FCM [40]. It is also not suited to identify novel phosphorylation sites or interactions. When using native conditions for the IP and general anti-phospho-tyrosine antibodies for the staining step (as in figure 2), one should consider that the antibody might probe all accessible phospho-tyrosines of the purified protein complex. For example, the phospho-tyrosine signal in figure 2b is a mixture of phospho-CD3 and associated phospho-proteins, such as phospho-ZAP70. If this is not desired, we recommend a denaturation step before the IP. Another potential drawback of IP-FCM is the fact that epitopes might be spatially blocked by bound proteins, conformational changes or covalent modifications. Again, denaturation could be of advantage, as we did when measuring phospho-Erk levels.

IP-FCM is best suited to generate large quantitative, multidimensional data sets on protein phosphorylations and interactions that are already known and for which good antibodies exist.

Sensitivity of IP-FCM might be enhanced by increasing the concentration of the lysate (lysis of cells in smaller volume), by reducing the number of beads used per sample, by increasing the concentration of the staining antibodies or by using a primary and secondary staining reagent, such as a biotinylated first antibody and fluorophore-coupled streptavidin.

Using multi-colour IP-FCM, we reconstructed with high quantitative accuracy the dynamics of phosphorylations at the TCR-CD3 and ZAP70, which have previously been partially characterized by IP-WB and one-colour IP-FCM [7], [41]. These data have allowed us to develop a mechanistic model of the underlying TCR-CD3-ZAP70 interaction and reversible phosphorylations and (unlike the IP-WB data with their large error bars) have forced the model to precisely reproduce the kinetics of ZAP70 recruitment and phosphorylation. As a result, the model has correctly predicted the temporal relation of two key ZAP70 phosphorylations, comparatively early phosphorylation of Y319 by Lck and delayed trans-autophosphorylation of Y493. Thus, an earlier suggestion that phosphorylation at Y319 required for Y493 phosphorylation [26], is enforced by our study.

Unexpectedly, our data also showed that pervanadate stimulation of the cells led to a transient decrease of the pY319-ZAP70/ZAP70 ratio at the TCR-CD3. Due to the large errors of IP-WB, this conclusion could not be drawn. Importantly, the mathematical model demonstrated that the initial massive recruitment of non-phosphorylated ZAP70 was responsible for this seemingly counter-intuitive kinetic behavior. Indeed, we could experimentally verify that a small amount of pY319-ZAP70 is pre-bound to the TCR-CD3 in resting cells and that the bulk of ZAP70 in the cytosol is in the non-phosphorylated state. Thus, recruitment of the cytosolic ZAP70 pool to the TCR-CD3 upon stimulation leads to a transient decrease of the pY319-ZAP70/ZAP70 ratio at the TCR-CD3. At later time points the ratio increases, due to phosphorylation of ZAP70 bound to the TCR-CD3.

The quantitative agreements between data and mathematical simulations corroborates the underlying mechanistic model, underscoring the need for *de novo* phosphorylation of ZAP70 recruited to the TCR-CD3, followed by trans-autophosphorylation of ZAP70 molecules. In conclusion, the high accuracy and sensitivity of IP-FCM is suited to elucidate the temporal coding of cell signalling events to unrivalled accuracy.

## Materials and Methods

### Ethics statement

Animal experiments were done in compliance with the guidelines of the German law and the Max-Planck-Institute for Immunobiology and Epigenetics (MPIIBE). The project was submitted and approved by the MPIIBE animal ethics board and registered with the Freiburg regional council (permit number: Re-iTO-5).

### Antibodies

Anti-TCRβ (597-H57, BD Pharmingen), anti-LAT (11B.12, BioLegend), anti-phospho-tyrosine (4G10, Upstate Biotechnology) or anti-Erk1/2 (7D8, abcam) monoclonal antibodies were covalently coupled to CML latex beads (Invitrogen) as described[8]. The following fluorophore-conjugated antibodies were used for staining: anti-CD3ε-APC (145-2C11, eBioscience), anti-ZAP70-alexa488 (1E.7.2 invitrogen), anti-phospho-ZAP70(Y319)-PE (17A/P-ZAP70, BD Biosciences), anti-phospho-tyrosine-PE (PY20, abcam), anti-Erk1/2-alexa488 (upstate biotechnology) and anti-phospho-Erk1/2-alexa647 (E10, cell signalling). For Western blot, anti-ZAP70 antibody (29/ZAP70 Kinase, BD Transduction Laboratories) and anti-GAPDH antibody (rabbit polyclonal, abcam) were purchased.

### Cells

The murine cell line 2B4 [33], [34] was maintained in complete RPMI 1640 medium supplemented with 5% fetal bovine serum [42]. OT-1 mice [35], which contain transgenes for the OVA-specific OT-1 TCRα and TCRβ chains were killed and single cell suspensions of spleen and thymus were prepared. Erythrocytes were lysed by incubating cells in 1 ml ACK erythrocyte lysis solution for two minutes. The ACK solution was removed by centrifugation and the remaining cell pellet was resuspended in RPMI 1640 medium without serum.

### Cell stimulation

Cells were stimulated in RPMI 1640 medium without serum at 20 million cells per sample, after incubation for 1 hr at 37°C prior to stimulation. Stimulation was done with either (i) 5 mM pervanadate, or (ii) a mixture of the anti-CD3ε antibody 145-2C11 and the anti-TCRβ antibody 597-H57 both at 5 µg/ml, or (iii) 100 nM H2K^b^-OVA MHC-peptide tetramers (Beckman Coulter) for different time points at 37°C. Cells were lysed in 1 ml lysis buffer containing 20 mM TrisHCl (pH 8), 137 mM NaCl, 2 mM EDTA, 10% glycerol, 10 mg/ml leupeptin, 10 mg/ml aprotinin, 1 mM PMSF, 500 mM sodium orthovanadate, 1 mM NaF and 0.3% Brij96 [43]. After 15 min incubation on ice, the insoluble material was removed by centrifugation.

### Immuno-precipitation measured by flow cytometry (IP-FCM)

30000 beads/sample were added to the lysate and 3 h or overnight IP was done by rotating at 4°C. After IP, immunoprecipitates were washed three times with blocking solution (phosphate buffer saline (PBS) containing 10% BSA, 0.5% tween and 0.05% sodium azide). Staining was done with different fluorophore-labelled monoclonal antibodies anti-CD3ε 1∶100, anti-Erk 1∶50, anti-pErk 1∶10, pTyr 1∶50, anti-ZAP70 1∶100 and anti-phospho-ZAP70 was a prediluted antibody. After incubation for 30 minutes on ice in the dark by rotation, beads were washed three times with blocking solution, resuspended in 150 µl of PBS and fluorescence of 5000 beads measured by FCM using the Gallios flow cytometer (Beckman coulter) or the FACSCalibur (Becton Dickinson) machines. The Gallios flow cytometer includes automation to facilitate high-throughput performance of multi-colour FCM assays. Flow cytometric data were analyzed using FlowJo (Tree Star, Inc.) and further statistical analysis was done using Prism GraphPad (Graphpad Software, Inc.).

In the modified protocol for normalized quantification of phosopho-Erk the following steps were modified: Staining with fluorescent anti-Erk and anti-phospho-Erk antibodies was not successful when Erk was captured in its native state using anti-Erk-coupled latex beads (data not shown). Most likely this was due to the fact that these antibodies normally are used in SDS-PAGE and Western blot experiments and thus, recognize the denatured, unfolded protein. We tested several conditions of denaturating the lysate without interfering with the antibodies used at later steps (**Fig. S6a**). Based on these results the best conditions were: addition of SDS to a final concentration of 0.3% to the lysate, boiling at 95°C for 5 min and no dilution of lysate for IP. The following IP and staining steps were done as described above.

A detailed description of one-colour IP-FCM method and a trouble-shooting section is published [7], [44].

### SDS-PAGE and Western blot analysis

IP was done using 2 µg of the respective antibody per sample and 10 µl of protein G-coupled sepharose beads (GE Healthcare Bio-Sciences AB) overnight by rotating at 4°C [45]. After IP, immunoprecipitates were washed three times with lysis buffer and boiled in reducing sample buffer at 95°C for 5 minutes. After separation of the samples by standard SDS-PAGE, semidry transfer was performed to PVDF membranes. Western blots were developed with the primary antibodies indicated and horseradish peroxidase (HRP)-conjugated secondary antibodies. Molecular weight standards were from Fermentas (pre-stained protein molecular weight marker). Detection and quantification of signals was done using Luminescent Image Analyzer LAS-4000 offered by Fujifilm Life Science. For quantification of the band intensities of ZAP70 and phospho-ZAP70, two separate gels were run from all replicates. One membrane from each replica was developed with anti-ZAP70 and anti-CD3ε and the other membrane with anti-phospho-ZAP70 and anti-CD3ε. Signal intensities of ZAP70 and phospho-ZAP70 were normalized with respect to their corresponding CD3ε values. For calculating the ratio of phospho-ZAP70/ZAP70, TCR normalized values of phospho-ZAP70 and ZAP70 were used.

### Quantibrite measurement

2B4 cells were stimulated with 5 mM pervanadate for different time points as in figure 1. IP was performed using anti-TCRβ antibody (H57) coupled beads overnight and the beads were divided in three equal aliquots. The aliquots were stained with anti-CD3ε-PE (145-2C11, BD Biosciences) or anti-ZAP70-PE (1E7.2, eBioscience) or anti-pZAP70-PE (17A/P-ZAP70, BD Biosciences) antibodies using a saturating concentration for staining (as determined in a pre-experiment by staining with different concentrations of each antibody). For comparison purpose, the same clone was used for each antibody as in figure 1, except that each antibody was labelled with PE. Standard PE-labelled Quantibrite beads from BD Biosciences were measured along with the samples to generate a standard curve of mean fluorescence intensity versus PE molecules per bead. The standard Quantibrite beads are a mixture of four beads coupled with 515, 5956, 26653 and 69045 PE molecules per bead. From the standard curve, PE molecules per bead for our samples were determined. By confirming the PE-labelling efficiency of each antibody used, respective molecules per bead were determined.

### Mathematical modeling

The mathematical model is based on the reaction scheme for a pair of opposing ITAMs (**Fig. 6**). For simplicity, we treat all ITAMs of the TCR complex as identical so that the fraction of recruited ZAP70 to an ITAM pair of the TCR equals the fraction of bound ZAP70 to the whole TCR complex. Although in reality, the different ITAMs of the paired ζ chains appear to show different behavior (e.g., the distal ITAMs might be phosphorylated more easily than the proximal ones), the simplified model turns out to provide an accurate fit to the experimental data and make verifiable predictions. Each ITAM can have seven states with respect to its tyrosine phosphorylation, ZAP70 recruitment, and ZAP70 phosphorylation at Y319 and at Y493. For a pair of opposite ITAMs, this results in 7×7 = 49 different states. As we treat phosphorylation of ITAM tyrosines as random and the two ITAMs as having identical properties, the model can be reduced to 28 states. Implementing first-order mass action kinetics, we obtain the following system of differential equations for the 28 distinguishable states:
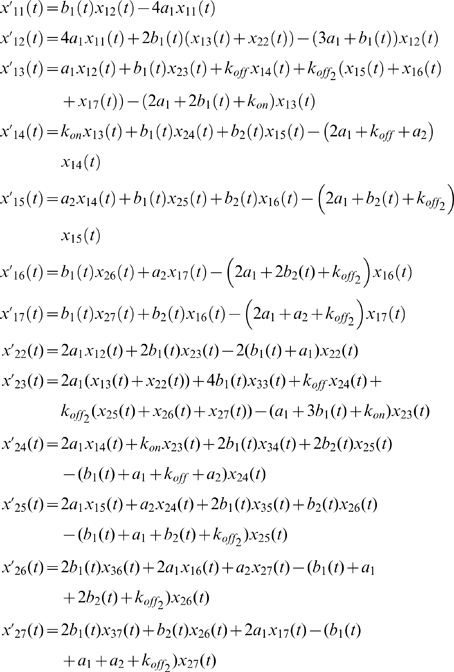


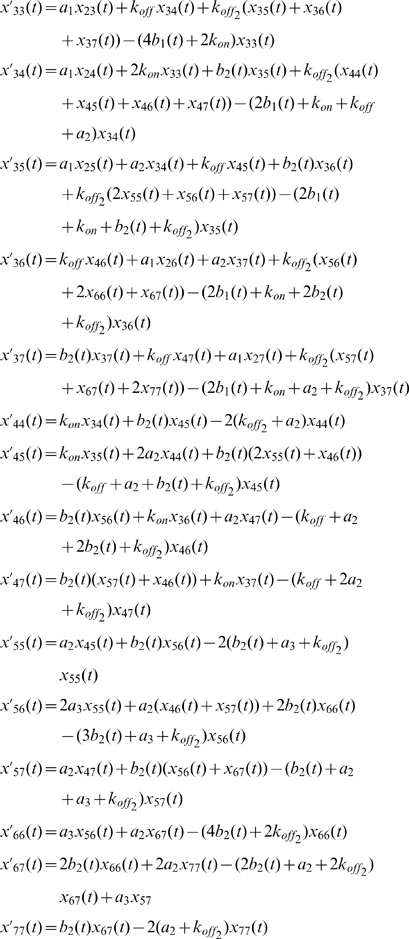



With 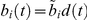.

To model inhibition of phosphatases by pervanadate, we decreased all rate constants of dephosphorylation. As phosphatase activity recovers with time (presumably due to ongoing phosphatase synthesis, see also figure 8), we accounted for a slow recovery of phosphatase activity by the function
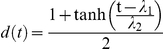
that multiplies all dephosphorylation rate constants *b_i_*. Prior to pervanadate stimulation, the model is in steady state with uninhibited phosphatase rate constants. All parameters were obtained by least-square fitting of the model simultaneously to the IP-FCM data for ZAP70 recruitment and Y319 phosphorylation, using a simulated annealing algorithm. For this purpose, the appropriate linear combinations of individual states *x_i_* (*t*), corresponding to ZAP70 bound to a doubly phosphorylated ITAM and bound pY319-ZAP70, were used. The parameters of the model are shown in figure S9.

## Supporting Information

Figure S1 **Protein concentration-dependence of the IP-FCM values.** 2B4 cells were left unstimulated (a) or stimulated with 5 mM pervanadate for 10 min (b). After cell lysis, the lysates were diluted to yield the different protein concentrations as indicated. After IP with 30000 anti-TCRβ-coupled latex beads, beads were stained with anti-CD3ε-APC and anti-ZAP70-alexa488 and fluorescence intensity measured by FCM. The geometric mean fluorescence intensity (MFI) is shown for APC (squares) and alexa488 (triangles). As expected, the more lysate was used, the stronger were the signals. Saturation was reached between 4 µg/ml and 8 µg/ml total protein in the lysate. ZAP70 recruitment was already detected with 0.4 µg/ml total protein concentration.
(TIF)

Figure S2 **Src kinase inhibition abrogates ZAP70 recruitment.** 2B4 cells were left untreated (black bars) or incubated with 20 µM of the Src-kinase inhibitor PP2 for 2 min (grey bars). Subsequently, cells were pervanadate-stimulated and three-color IP-FCM was performed as in figure 1. A significant decrease in ZAP70 and phospho-ZAP70 intensities per TCR-CD3 was observed upon PP2 treatment compared to untreated samples. This confirms the signalling dependent recruitment and phosphorylation of ZAP70.
(TIF)

Figure S3 **The anti-ZAP70 antibody 1E7.2 recognizes stimulated and unstimulated ZAP70.** 2B4 cells (a) or OT-1 splenocytes (b) were stimulated with the pervanadate or pMHC tetramers, respectively. Cells were lysed and immunoprecipitation of ZAP70 was performed using the 1E7.2 antibody under native conditions. After the IP, ZAP70 was detected by SDS-PAGE and Western blotting using the antibody clone 29/ZAP70 Kinase (BD Transduction Laboratories). As a control for the amount of cells, a WB of the lysates was developed for actin. In case of the 2B4 cells, an anti-phosphotyrosine (clone 4G10) development shows that the stimulation has worked. In case of the OT-1 splenocytes, stimulation was also successful, since the lysates used in this experiment were the same as the ones used for figure 3c and 3d. A similar amount of ZAP70 could be immunoprecipitated from the stimulated or non-stimulated cells, indicating that the 1E7.2 antibody can recognize stimulated and unstimulated ZAP70.
(TIF)

Figure S4 **Two-plexed IP-FCM.** Distinction of 3 µm from 10 µm latex beads in FCM. The dot plots representing forward and side scatter for 3 µm (left panel) and 10 µm (middle panel) beads or a mixture of both (right panel) are shown. When used in combination (mixed before IP in a 1∶1 ratio), 3 and 10 µm beads were analyzed separately based on the gating shown in pink lines after the flow cytometric measurement. The population marked with an asterisk in the left and right panels most likely corresponds to dimers of the 3 µm beads. These dot plots are taken from the two-color IP-FCM experiment shown in figure 2d, where the 3 µm beads were coupled to anti-TCRβ antibodies and the 10 µm beads to anti-LAT antibodies.
(TIF)

Figure S5 **Two-color IP-FCM.** 2B4 cells were stimulated with 5 mM pervanadate for 5 minutes (grey bars) or left unstimulated (black bars) and lysed. The lysate was denatured by boiling in 0.33% SDS at 95°C for 5 minutes. Anti-phospho-tyrosine IP was followed by simultaneous staining with anti-CD3ε-APC and anti-ZAP70-alexa488 antibodies. MFI of both fluorophores is shown (each is normalized to its unstimulated value). Hence the phosphorylation of different signalling proteins can be quantified in a multi-plex way by IP-FCM using anti-phospho-tyrosine antibody coupled beads for IP and staining with different fluorophore-labelled antibodies against proteins of interest.
(TIF)

Figure S6 **Optimization of denaturation conditions and lysate dilution for phospho-Erk measurements.** (a) 2B4 cells were stimulated with 5 µg/ml anti-TCRβ plus 5 µg/ml anti-CD3ε antibodies for 5 min or left unstimulated. Lysates were boiled in the indicated concentration of SDS at 95°C for 5 minutes and the indicated dilution of the boiled lysate was made using 0.3% Brij96 lysis buffer prior to performing IP with anti-Erk coupled beads. Anti-Erk-alexa488 and anti-phospho-Erk-alexa647 staining was done and measured with FCM. Fold change as calculated by dividing the normalized (with respect to total Erk) geometric mean fluorescence intensity (MFI) of phospho-Erk in the stimulated sample by that of the unstimulated sample is plotted in the histogram. The maximum fold change was seen in the condition of 0.33% SDS boiling and no dilution of lysate and was selected as best condition for further experiments. (b) Lysates from 2B4 cells stimulated with 5 mM pervanadate for 5 minutes or left unstimulated were boiled in 0.33% SDS at 95°C for 5 minutes and used for IP with anti-Erk antibody coupled beads. The beads were stained with the mentioned dilutions of labelled anti-phospho-Erk-alexa647. A larger difference in phospho-Erk intensity of stimulated versus unstimulated samples was observed, if higher concentrations of the staining antibody were used.
(TIF)

Figure S7 **Kinetics of Erk phophorylation measured with the commercially available BioPlex kit.** (a) 2B4 cells were stimulated with anti-TCRβ and anti-CD3ε antibodies for the indicated time points. Cells were lysed at a concentration of 2×10^7^ cells/ml lysis buffer. 50 µl of this lysate (corresponding to 0.2 µg total protein) was taken for overnight IP with 2500 anti-Erk antibody-coupled BioPlex beads. Then beads were stained using a biotin-coupled anti-phospho-Erk antibody and PE-labelled streptavidin. Measurements were done using the BioPlex instrument. The MFI of the anti-phospho-Erk staining is shown. The value was not normalized to total Erk as two-color staining is not possible with the BioPlex system. A clear increase in the phosphorylation of Erk was seen. (b) To test the sensitivity of the method, the following dilutions of the lysate from (a) were prepared: undiluted (200 ng), 1∶5 (40 ng) and 1∶10 (20 ng of total protein). These were used for IP with anti-Erk antibody-coupled BioPlex beads as in (a) and staining as in (a). The phospho-Erk intensity for different time points is shown for each of the lysate dilutions. The highest values for the phospho-Erk intensity were obtained with 40 and 20 ng of total protein. Thus, the BioPlex system is more sensitive than the two-color IP-FCM shown in figure 2e. This might be explained by the fact that a biotin-labelled primary antibody was used in BioPlex and signals were amplified using PE-labelled streptavidin. The two-step staining procedure might help in amplification of signals also for IP-FCM. Data collection by BioPlex. The vendor-provided protocol was followed for BioPlex measurement of Erk phosphorylation. Briefly, 2B4 cells were stimulated with anti-TCRβ and anti-CD3ε antibodies as for IP-FCM. Cells were lysed in lysis buffer (provided in the Bio-Rad Cell Lysis Kit) at a concentration of 20 million cells/ml. From this lysate, three dilutions were prepared for each sample to check the effect of protein concentration on BioPlex measurement. 50 µl of final dilution was used for each sample. After overnight IP with 2500 anti-Erk antibody coupled BioPlex beads/sample, staining was done according to the protocol using a biotin-coupled anti-phosphoErk antibody and PE-labelled streptavidin. Measurements were done using the BioPlex instrument. Only 25 beads were counted per sample (according to the user manual) and data were analysed using the BioPlex Manager™ software.
(TIF)

Figure S8 **Measurement of Quantibrite™** **beads for absolute quantification.** Along with the IP-FCM samples, standard calibration beads (Quantibrite™ beads from BD Biosciences) with a known number of PE molecules per bead were measured. (a) A histogram of the PE fluorescence of the beads is shown together with the exact number of PE molecules per bead (on top of each bead population. (b) The standard curve of log PE molecules per bead versus log MFI was generated to calculate the number of PE molecules on IP-FCM beads. A correlation coefficient close to 1 was obtained between log PE molecules per bead and log MFI.
(TIF)

Figure S9 **ZAP70 does not dissociate significantly from the TCR-CD3 complex during the immunoprecipitation and staining procedure.** 2B4 cells were stimulated with 5 mM pervanadate for 10 minutes or left unstimulated and the immunoprecipitation with anti-TCR-coupled latex beads and three-color IP-FCM using anti-CD3-APC, anti-ZAP70-alexa488 and anti-pY319-ZAP70-PE was performed for different durations as follows. IP was done either for 2 h or 6 h and the beads were stained with the antibodies for 45 min. Including cell lysis and washing the procedures took 3 h and 7 h. In addition, after the 6 h IP beads were stained for 12 h (overnight) with the staining antibodies, resulting in a total procedure of approx. 18 h. The beads were measured at the end of each procedure using the same cytometer with the same instrument settings. The ZAP70/CD3ε ratio was the same for each procedure (non-paired t-test between each group, panel a). Error bars represent mean +/− s.e.m., n = 3. To control that the recordings were done with the same sensitivity, each of the three measurements (3 h, 7 h and 18 h) was complemented with a measurement of the four different PE-labelled quantibrite calibration beads (b). Indeed, the geometric mean fluorescence intensity of the corresponding quantibrite beads was the same in each of the three measurements. In conclusion, ZAP70 does not dissociate from the TCR-CD3 complex even after an 18 hours IP and staining procedure.
(TIF)

Figure S10 **Parameters of the mathematical model.** The parameters of our model, which is shown in figure 6 and given in Material and Methods, are given.
(TIF)

Table S1 **Statistical analysis of the accuracy of IP-FCM compared to the one of WB.**
(DOC)
