# Supplementary figures and images for: Quantitative Analysis of Protein Phosphorylations and Interactions by Multi-Colour IP-FCM as an Input for Kinetic Modelling of Signalling Networks
Source: PLoS One. 2011 Jul 29;6(7):e22928. doi: 10.1371/journal.pone.0022928 (PMC3146539; doi:10.1371/journal.pone.0022928)

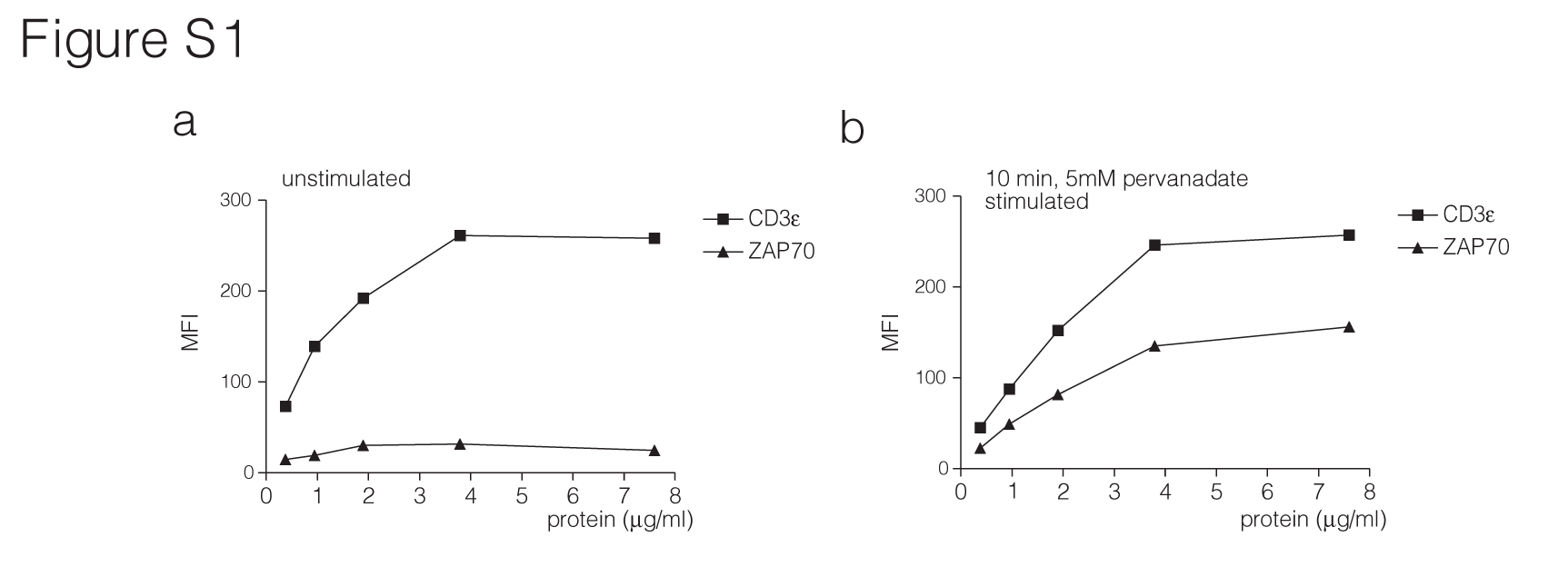

Supplement: Figure S1 — Protein concentration-dependence of the IP-FCM values. 2B4 cells were left unstimulated (a) or stimulated with 5 mM pervanadate for 10 min (b). After cell lysis, the lysates were diluted to yield the different protein concentrations as indicated. After IP with 30000 anti-TCRβ-coupled latex beads, beads were stained with anti-CD3ε-APC and anti-ZAP70-alexa488 and fluorescence intensity measured by FCM. The geometric mean fluorescence intensity (MFI) is shown for APC (squares) and alexa488 (triangles). As expected, the more lysate was used, the stronger were the signals. Saturation was reached between 4 µg/ml and 8 µg/ml total protein in the lysate. ZAP70 recruitment was already detected with 0.4 µg/ml total protein concentration. (TIF) [file pone.0022928.s001.tif]

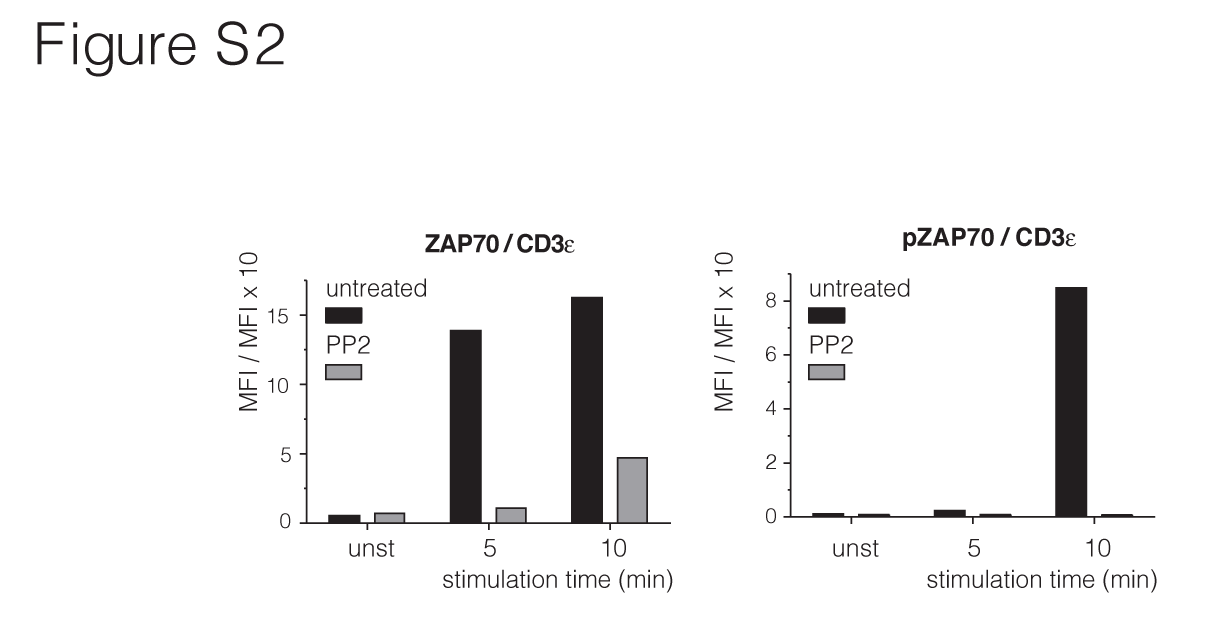

Supplement: Figure S2 — Src kinase inhibition abrogates ZAP70 recruitment. 2B4 cells were left untreated (black bars) or incubated with 20 µM of the Src-kinase inhibitor PP2 for 2 min (grey bars). Subsequently, cells were pervanadate-stimulated and three-color IP-FCM was performed as in figure 1. A significant decrease in ZAP70 and phospho-ZAP70 intensities per TCR-CD3 was observed upon PP2 treatment compared to untreated samples. This confirms the signalling dependent recruitment and phosphorylation of ZAP70. (TIF) [file pone.0022928.s002.tif]

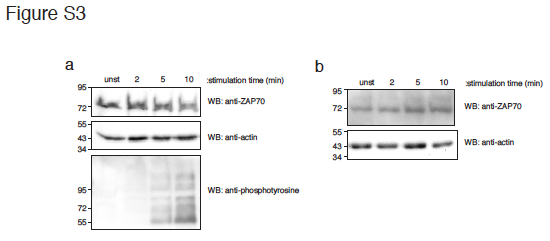

Supplement: Figure S3 — The anti-ZAP70 antibody 1E7.2 recognizes stimulated and unstimulated ZAP70. 2B4 cells (a) or OT-1 splenocytes (b) were stimulated with the pervanadate or pMHC tetramers, respectively. Cells were lysed and immunoprecipitation of ZAP70 was performed using the 1E7.2 antibody under native conditions. After the IP, ZAP70 was detected by SDS-PAGE and Western blotting using the antibody clone 29/ZAP70 Kinase (BD Transduction Laboratories). As a control for the amount of cells, a WB of the lysates was developed for actin. In case of the 2B4 cells, an anti-phosphotyrosine (clone 4G10) development shows that the stimulation has worked. In case of the OT-1 splenocytes, stimulation was also successful, since the lysates used in this experiment were the same as the ones used for figure 3c and 3d. A similar amount of ZAP70 could be immunoprecipitated from the stimulated or non-stimulated cells, indicating that the 1E7.2 antibody can recognize stimulated and unstimulated ZAP70. (TIF) [file pone.0022928.s003.tif]

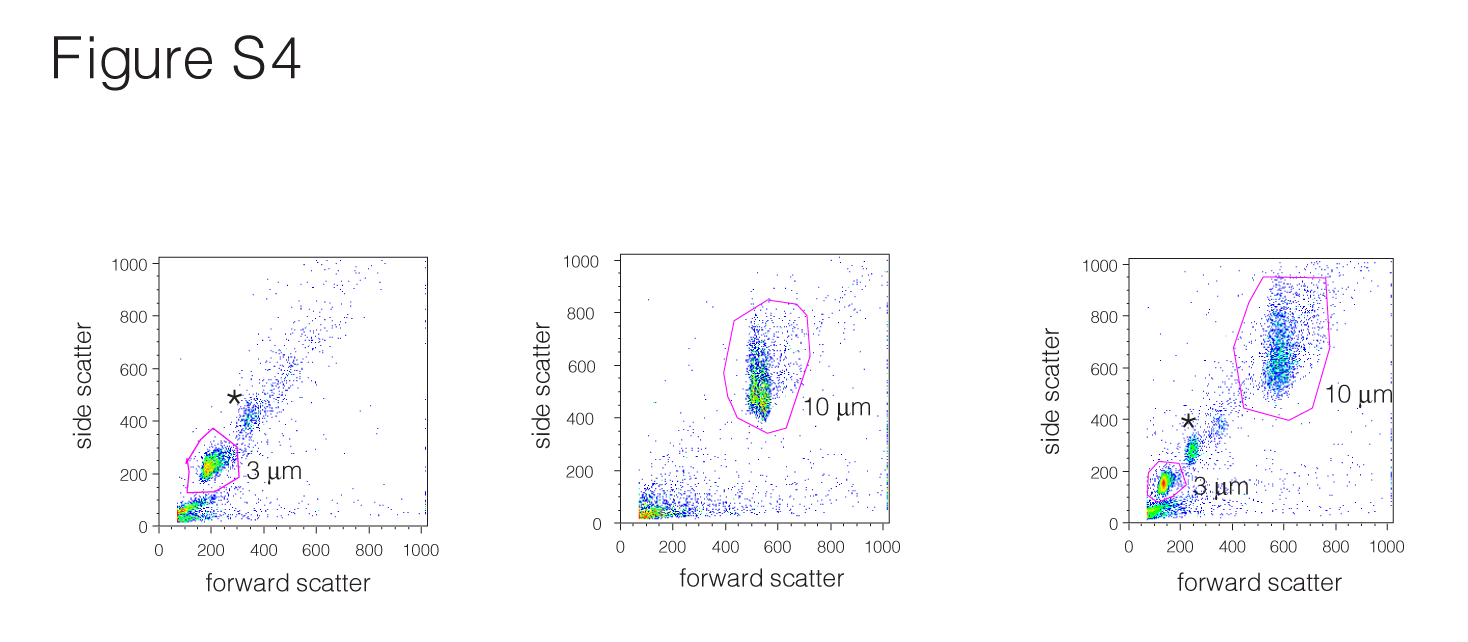

Supplement: Figure S4 — Two-plexed IP-FCM. Distinction of 3 µm from 10 µm latex beads in FCM. The dot plots representing forward and side scatter for 3 µm (left panel) and 10 µm (middle panel) beads or a mixture of both (right panel) are shown. When used in combination (mixed before IP in a 1∶1 ratio), 3 and 10 µm beads were analyzed separately based on the gating shown in pink lines after the flow cytometric measurement. The population marked with an asterisk in the left and right panels most likely corresponds to dimers of the 3 µm beads. These dot plots are taken from the two-color IP-FCM experiment shown in figure 2d, where the 3 µm beads were coupled to anti-TCRβ antibodies and the 10 µm beads to anti-LAT antibodies. (TIF) [file pone.0022928.s004.tif]

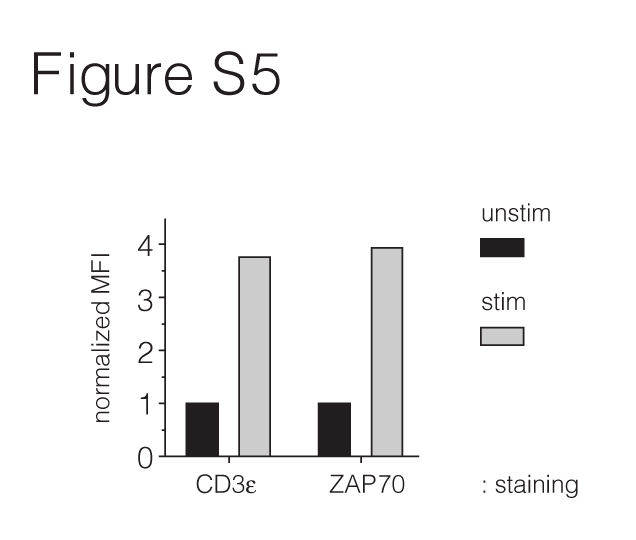

Supplement: Figure S5 — Two-color IP-FCM. 2B4 cells were stimulated with 5 mM pervanadate for 5 minutes (grey bars) or left unstimulated (black bars) and lysed. The lysate was denatured by boiling in 0.33% SDS at 95°C for 5 minutes. Anti-phospho-tyrosine IP was followed by simultaneous staining with anti-CD3ε-APC and anti-ZAP70-alexa488 antibodies. MFI of both fluorophores is shown (each is normalized to its unstimulated value). Hence the phosphorylation of different signalling proteins can be quantified in a multi-plex way by IP-FCM using anti-phospho-tyrosine antibody coupled beads for IP and staining with different fluorophore-labelled antibodies against proteins of interest. (TIF) [file pone.0022928.s005.tif]

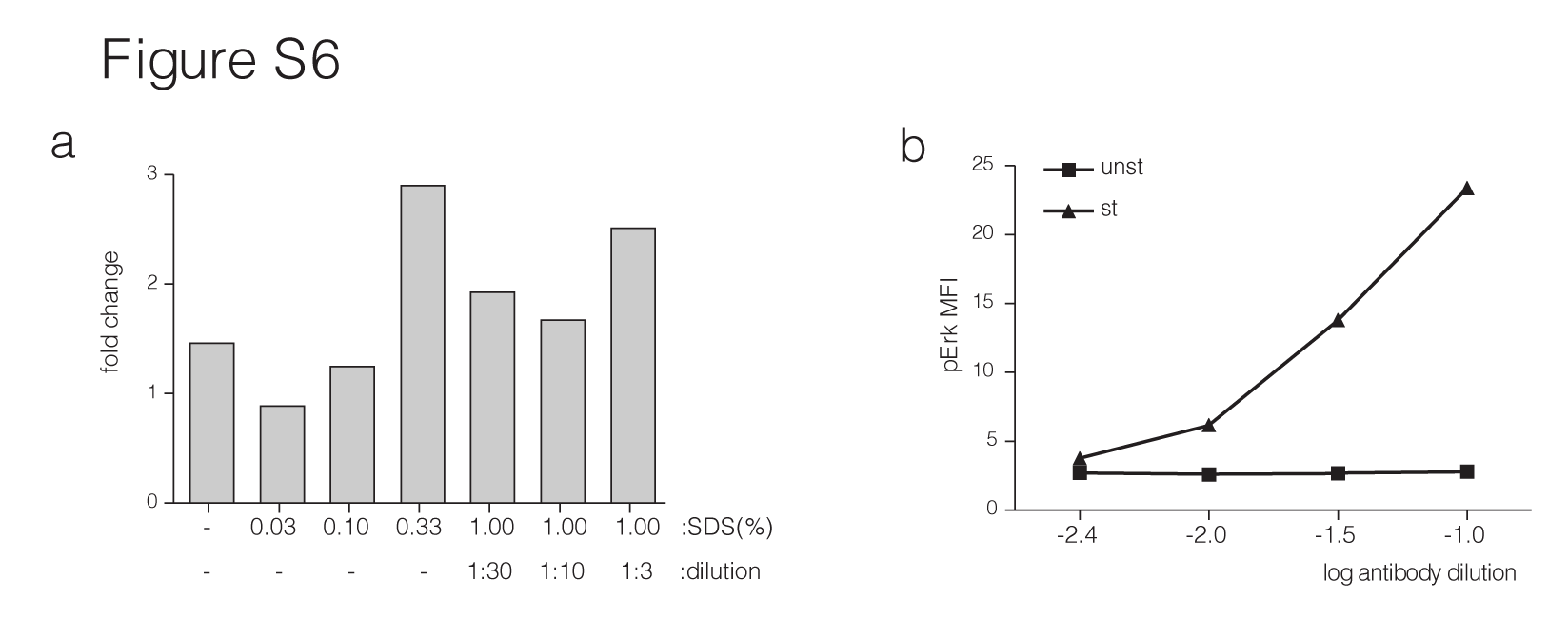

Supplement: Figure S6 — Optimization of denaturation conditions and lysate dilution for phospho-Erk measurements. (a) 2B4 cells were stimulated with 5 µg/ml anti-TCRβ plus 5 µg/ml anti-CD3ε antibodies for 5 min or left unstimulated. Lysates were boiled in the indicated concentration of SDS at 95°C for 5 minutes and the indicated dilution of the boiled lysate was made using 0.3% Brij96 lysis buffer prior to performing IP with anti-Erk coupled beads. Anti-Erk-alexa488 and anti-phospho-Erk-alexa647 staining was done and measured with FCM. Fold change as calculated by dividing the normalized (with respect to total Erk) geometric mean fluorescence intensity (MFI) of phospho-Erk in the stimulated sample by that of the unstimulated sample is plotted in the histogram. The maximum fold change was seen in the condition of 0.33% SDS boiling and no dilution of lysate and was selected as best condition for further experiments. (b) Lysates from 2B4 cells stimulated with 5 mM pervanadate for 5 minutes or left unstimulated were boiled in 0.33% SDS at 95°C for 5 minutes and used for IP with anti-Erk antibody coupled beads. The beads were stained with the mentioned dilutions of labelled anti-phospho-Erk-alexa647. A larger difference in phospho-Erk intensity of stimulated versus unstimulated samples was observed, if higher concentrations of the staining antibody were used. (TIF) [file pone.0022928.s006.tif]

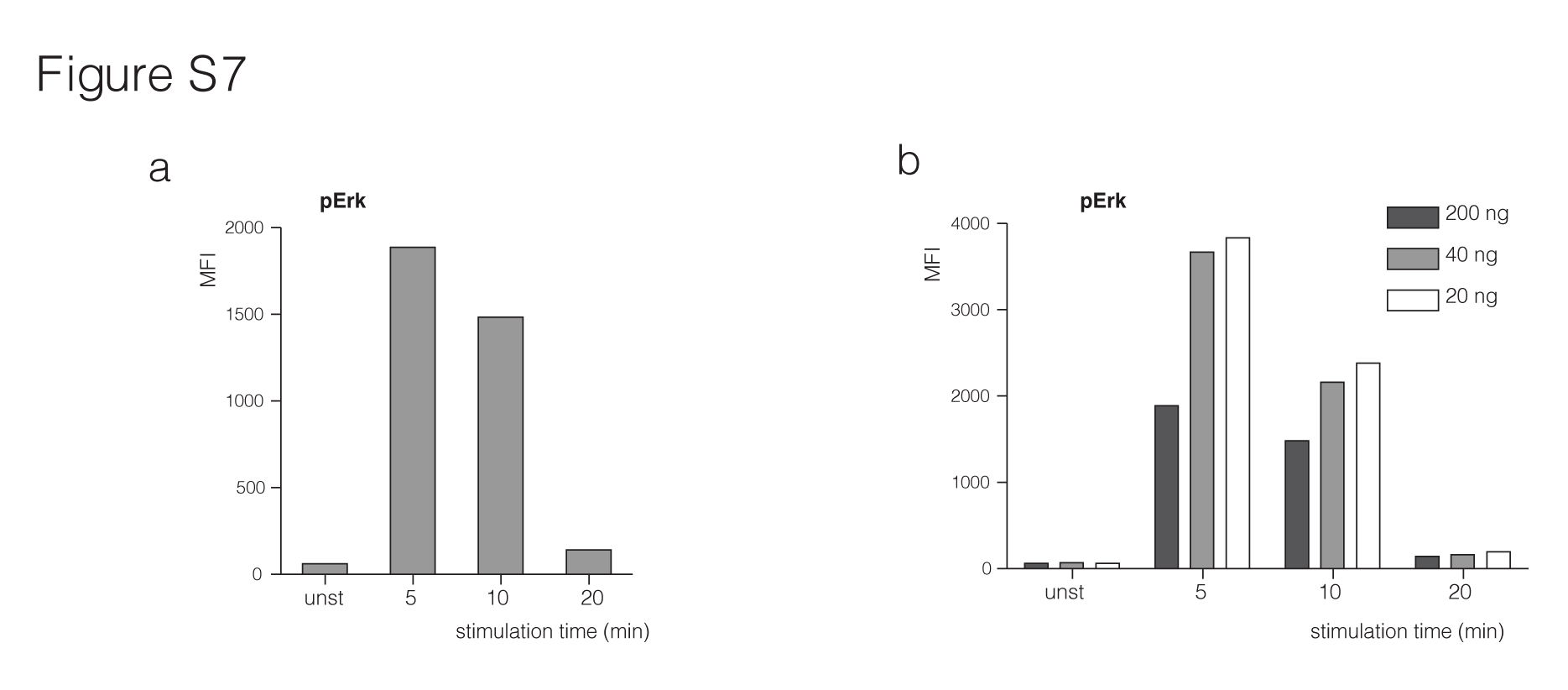

Supplement: Figure S7 — Kinetics of Erk phophorylation measured with the commercially available BioPlex kit. (a) 2B4 cells were stimulated with anti-TCRβ and anti-CD3ε antibodies for the indicated time points. Cells were lysed at a concentration of 2×10^7^ cells/ml lysis buffer. 50 µl of this lysate (corresponding to 0.2 µg total protein) was taken for overnight IP with 2500 anti-Erk antibody-coupled BioPlex beads. Then beads were stained using a biotin-coupled anti-phospho-Erk antibody and PE-labelled streptavidin. Measurements were done using the BioPlex instrument. The MFI of the anti-phospho-Erk staining is shown. The value was not normalized to total Erk as two-color staining is not possible with the BioPlex system. A clear increase in the phosphorylation of Erk was seen. (b) To test the sensitivity of the method, the following dilutions of the lysate from (a) were prepared: undiluted (200 ng), 1∶5 (40 ng) and 1∶10 (20 ng of total protein). These were used for IP with anti-Erk antibody-coupled BioPlex beads as in (a) and staining as in (a). The phospho-Erk intensity for different time points is shown for each of the lysate dilutions. The highest values for the phospho-Erk intensity were obtained with 40 and 20 ng of total protein. Thus, the BioPlex system is more sensitive than the two-color IP-FCM shown in figure 2e. This might be explained by the fact that a biotin-labelled primary antibody was used in BioPlex and signals were amplified using PE-labelled streptavidin. The two-step staining procedure might help in amplification of signals also for IP-FCM. Data collection by BioPlex. The vendor-provided protocol was followed for BioPlex measurement of Erk phosphorylation. Briefly, 2B4 cells were stimulated with anti-TCRβ and anti-CD3ε antibodies as for IP-FCM. Cells were lysed in lysis buffer (provided in the Bio-Rad Cell Lysis Kit) at a concentration of 20 million cells/ml. From this lysate, three dilutions were prepared for each sample to check the effect of protein concentration on B [file pone.0022928.s007.tif]

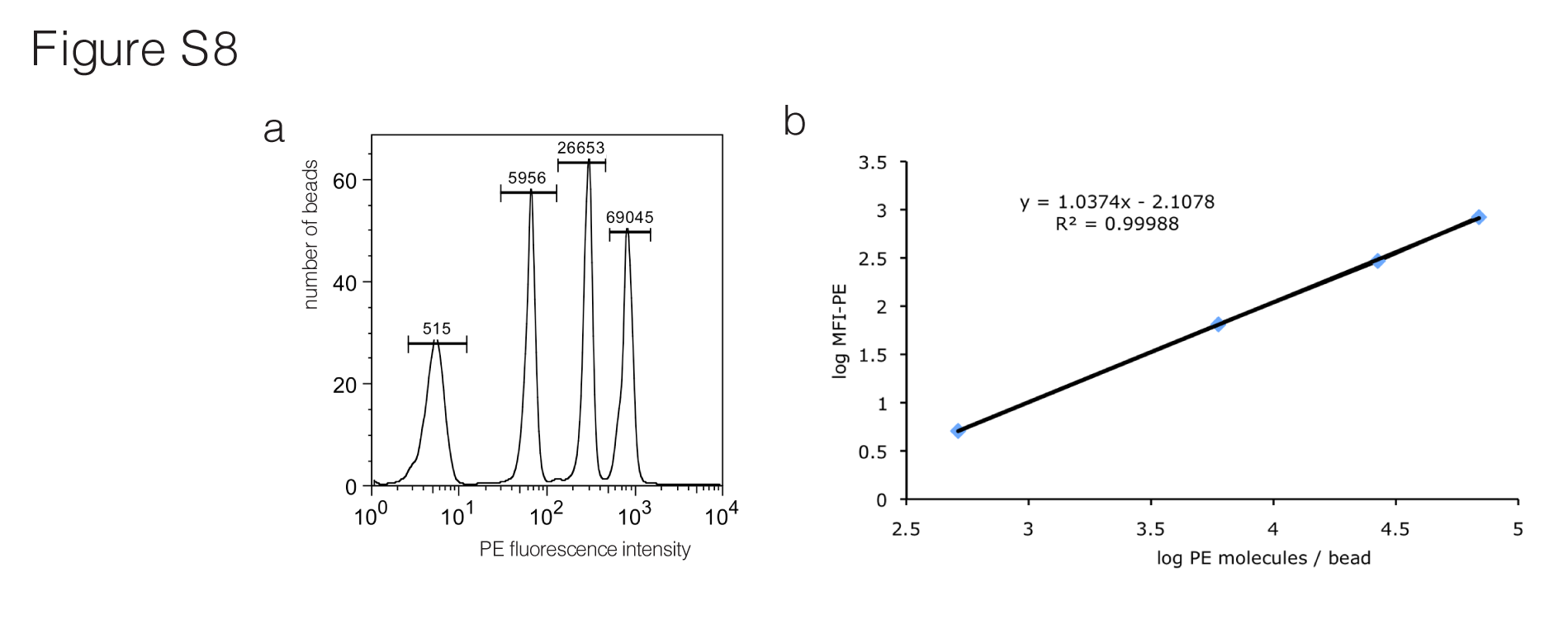

Supplement: Figure S8 — Measurement of Quantibrite™ beads for absolute quantification. Along with the IP-FCM samples, standard calibration beads (Quantibrite™ beads from BD Biosciences) with a known number of PE molecules per bead were measured. (a) A histogram of the PE fluorescence of the beads is shown together with the exact number of PE molecules per bead (on top of each bead population. (b) The standard curve of log PE molecules per bead versus log MFI was generated to calculate the number of PE molecules on IP-FCM beads. A correlation coefficient close to 1 was obtained between log PE molecules per bead and log MFI. (TIF) [file pone.0022928.s008.tif]

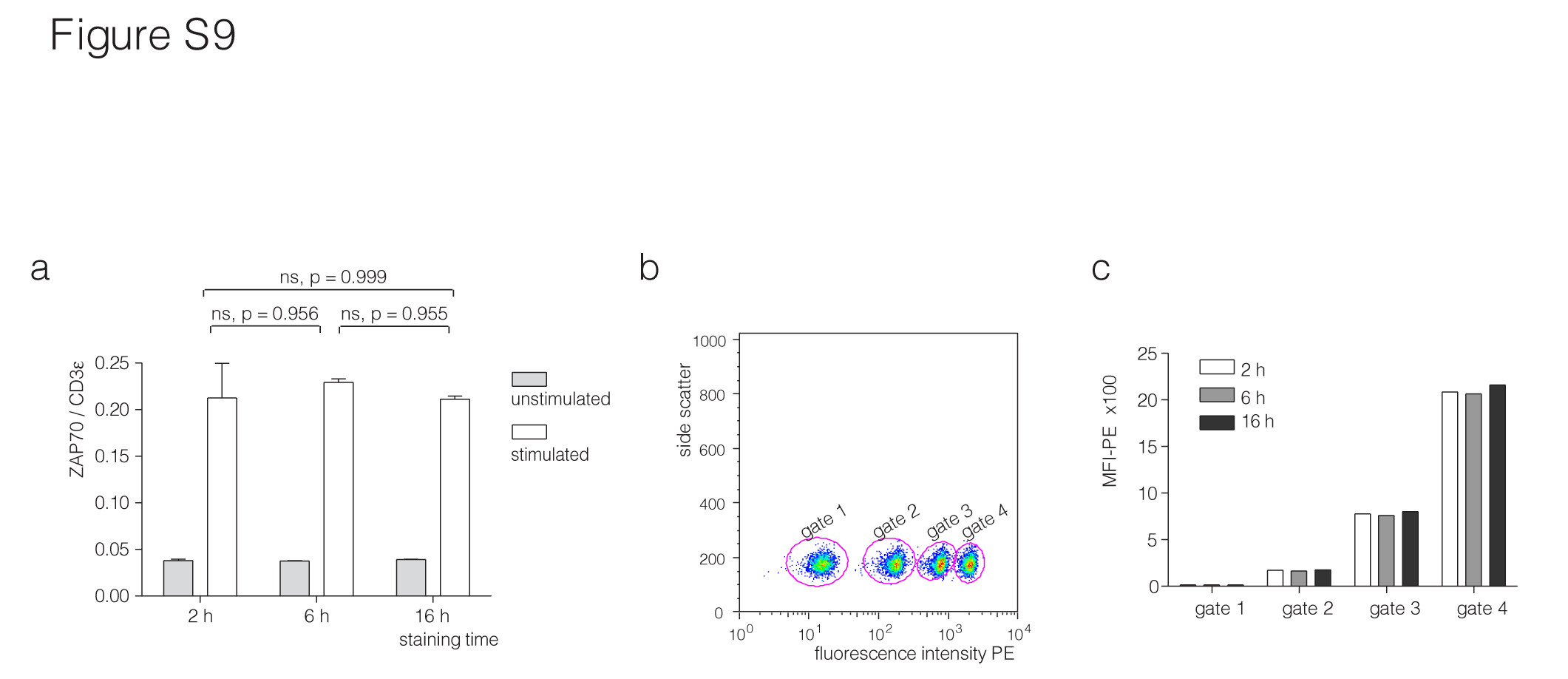

Supplement: Figure S9 — ZAP70 does not dissociate significantly from the TCR-CD3 complex during the immunoprecipitation and staining procedure. 2B4 cells were stimulated with 5 mM pervanadate for 10 minutes or left unstimulated and the immunoprecipitation with anti-TCR-coupled latex beads and three-color IP-FCM using anti-CD3-APC, anti-ZAP70-alexa488 and anti-pY319-ZAP70-PE was performed for different durations as follows. IP was done either for 2 h or 6 h and the beads were stained with the antibodies for 45 min. Including cell lysis and washing the procedures took 3 h and 7 h. In addition, after the 6 h IP beads were stained for 12 h (overnight) with the staining antibodies, resulting in a total procedure of approx. 18 h. The beads were measured at the end of each procedure using the same cytometer with the same instrument settings. The ZAP70/CD3ε ratio was the same for each procedure (non-paired t-test between each group, panel a). Error bars represent mean +/− s.e.m., n = 3. To control that the recordings were done with the same sensitivity, each of the three measurements (3 h, 7 h and 18 h) was complemented with a measurement of the four different PE-labelled quantibrite calibration beads (b). Indeed, the geometric mean fluorescence intensity of the corresponding quantibrite beads was the same in each of the three measurements. In conclusion, ZAP70 does not dissociate from the TCR-CD3 complex even after an 18 hours IP and staining procedure. (TIF) [file pone.0022928.s009.tif]

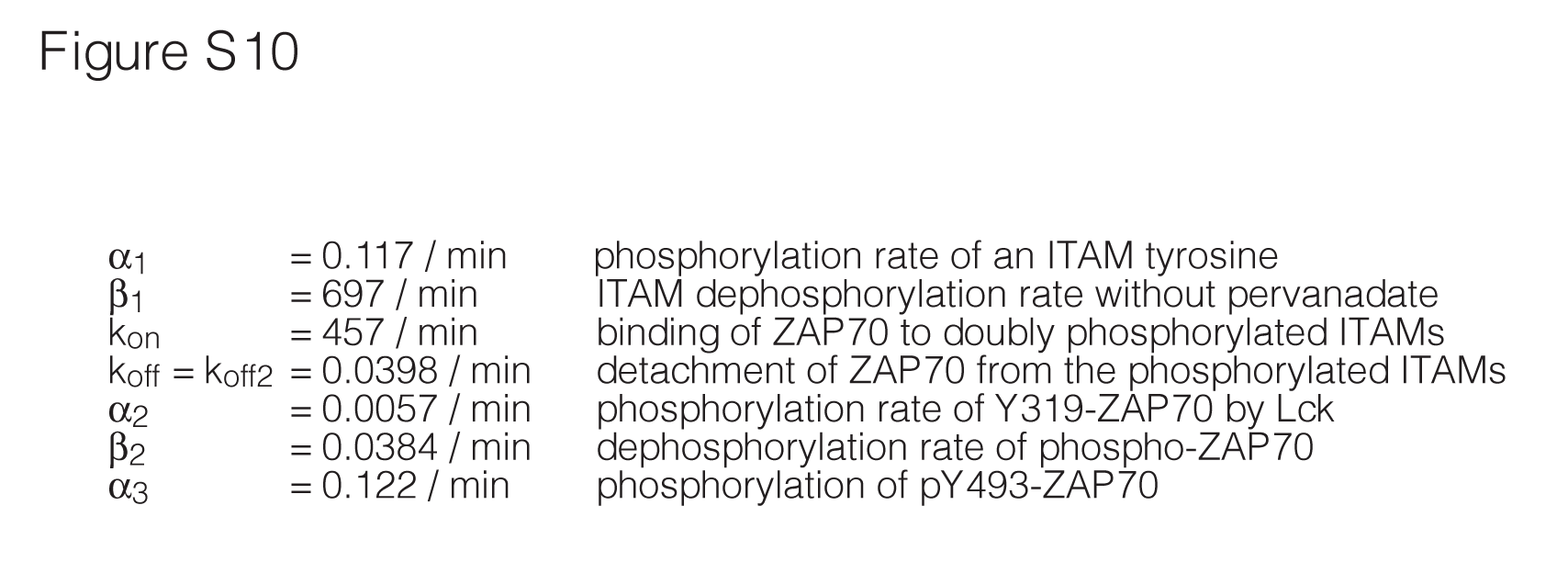

Supplement: Figure S10 — Parameters of the mathematical model. The parameters of our model, which is shown in figure 6 and given in Material and Methods, are given. (TIF) [file pone.0022928.s010.tif]
