# Supplementary material for: Quantitative Analysis of Protein Phosphorylations and Interactions by Multi-Colour IP-FCM as an Input for Kinetic Modelling of Signalling Networks
Source: PLoS One. 2011 Jul 29;6(7):e22928. doi: 10.1371/journal.pone.0022928 (PMC3146539; doi:10.1371/journal.pone.0022928)
Supplement: Table S1 — Statistical analysis of the accuracy of IP-FCM compared to the one of WB. (DOC) [file pone.0022928.s011.doc]

**Table S1**

Statistical analysis of the accuracy of IP-FCM compared to the one of WB.

| Parameter | Average %cv - WB* | Average %cv - IP-FCM |
| --- | --- | --- |
| ZAP70 | 38.5 | 5.5 |
| pZAP70 | 85.8 | 7.2 |
| TCR | 30.5 | 4.0 |
| Average of the above 3 parameters | 51.6 | 5.6 |

* The % coefficient of variation (%cv) in the MFI for IP-FCM and the signal intensity for WB of ZAP70, pZAP70 and TCR was calculated for each of the six stimulation time points shown in figures 1c and e. Average from %cv of these six stimulation time points is shown in the table for each of the three parameters measured. Average of average %cv for all the three parameters was calculated and found to be approximately 10 times higher (51.6) for WB compare to IP-FCM (5.6).
